# Pantry clients and Supplemental Nutrition Assistance Program-Education practitioners’ perspectives on factors influencing healthy eating policy, system and environmental interventions in food pantries

**DOI:** 10.1017/jns.2023.64

**Published:** 2023-07-19

**Authors:** Owusua Yamoah, Jillian Schulte, Lindsay Osborn, Callie Ogland-Hand, Ana Claudia Zubieta, Darcy A. Freedman

**Affiliations:** 1 Mary Ann Swetland Center for Environmental Health, School of Medicine, Case Western Reserve University, 10900 Euclid Avenue, Cleveland, OH 44106, USA; 2 Department of Population and Quantitative Health Sciences, School of Medicine, Case Western Reserve University, 10900 Euclid Avenue, Cleveland, OH 44106, USA; 3 Department of Anthropology, Case Western Reserve University, 10900 Euclid Avenue, Cleveland, OH 44106, USA; 4 College of Food, Agricultural, and Environmental Sciences (CFAES), OSU Extension, 381B Campbell Hall, 1787 Neil Avenue, Columbus, OH 43210, USA

**Keywords:** Assessment, Food pantry, Healthy eating, Policy, systems, and environmental (PSE) interventions, Readiness, COREQ, the consolidated criteria for reporting qualitative research, IRB, Institutional Review Board, NPP, Nutrition Pantry Program, OSU, Ohio State University, PSE, policy, system and environmental, READI, Readiness Assessment and Decision Instrument, RUCA, Rural–Urban Commuting Area, SNAP, Supplemental Nutrition Assistance Program, SNAP-Ed, Supplemental Nutrition Assistance Program Education program, WIC, Special Supplemental Nutrition Program for Women, Infants and Children

## Abstract

The Supplemental Nutrition Assistance Program-Education identified food pantries as a targeted setting for policy, system, and environmental (PSE) interventions to promote healthy eating among households who rely on pantries to supplement their food needs. The present study sought to identify factors influencing capacity and readiness to implement healthy eating PSE interventions in food pantries. Qualitative interviews were conducted via zoom with twenty-six community residents with experience receiving SNAP benefits and twelve SNAP-Ed staff in rural and urban counties in Ohio to identify themes and indicators related to community/organisational capacity and readiness to implement healthy eating PSE interventions in food pantries. Themes and related indicators generated based on inductive and deductive coding of interview transcripts were prioritised and weighted by eleven community nutrition experts during a virtual consensus conference. Five themes emerged; expert-derived weights (scaled low, 0 to high, 1) reflect the perceived importance of each to implementation of healthy eating PSE interventions in food pantries: food pantry capacity and logistics [0⋅252], networks and relationships [0⋅228], community nutrition practitioner capacity [0⋅212], food pantry user characteristics [0⋅156], and stigma and stereotypes [0⋅1⋅52]. Overall, seventeen indicators were identified reflecting these themes. Successful and sustained PSE interventions at food pantries will require a tailored approach that considers food pantries’ capacity, needs and opportunities within the community, and capacity of community nutrition practitioners. The themes and indicators identified provide guidance for responsive PSE approaches in food pantries that meet communities where they are.

## Introduction

Over 10 % of the U.S. population is food insecure^(1)^. Those with food insecurity do not consistently have enough quality food for all members of their household and often rely on the Supplemental Nutrition Assistance Program (SNAP) and emergency food assistance programs as a safety net to fill nutritional and food budget gaps^(2–4)^. Food pantries are part of the emergency food assistance network involved in providing free food and other supplies to households across the country. When examining food insecure households specifically, 36⋅5 % reported using a food pantry, emphasising the importance of these safety net programs^(2)^.

Recent economic crises resulting from the COVID-19 pandemic significantly increased reliance on food pantries across the country^(5)^. In 2020, over 6 % of all households in the United States utilised food pantries compared to 4⋅4 % in 2019^(2)^. Research by Caspi *et al*. found that over half of those who use food pantries receive 50 % or more of their household food from these pantries^(5)^. However, a systematic review found that emergency food distribution sites, including food pantries, often provide food with limited nutritional value^(6)^. This heavy reliance on food pantries, paired with existing evidence relating to poor diet, is at the root of the increased attention to healthy eating policy, system, and environmental (PSE) interventions in food pantries^(7)^.

A range of PSE interventions have been implemented at food pantries throughout the country to support healthy eating and improve nutritional benefits^(8–12)^. Examples of these interventions include increasing healthy food options within the pantry^(10,11)^ and implementation of client choice models to enhance the selection of healthier food options^(8,12)^. Although some of these interventions have been deemed successful, many do not consider the unique readiness and capacity of each pantry and community^(5,8)^. For instance, the Nutrition Pantry Program (NPP), a PSE change intervention that includes pantry and clients’ assessments does not account for the overall systems and environment within which pantries operate^(13)^. Variations in logistics within pantry sites, as well as community and clientele food preferences, may hinder PSE implementation and sustainability in these settings^(11,14)^.

There are limited tools available that consider variability of readiness and capacity within food pantry environments, thus highlighting an important gap to be addressed^(15)^. Implementation science has demonstrated that community-tailored approaches are necessary to fully consider the barriers, strengths, capacities and interests of *specific* communities and settings to maximise PSE impact^(16,17)^. Given the importance of food pantries to food insecure populations, scholars^(18)^, as well as organisations like the SNAP Education program (SNAP-Ed)^(19)^, have identified food pantries as a targeted setting for tailored PSE interventions. In the present paper, we highlight findings of a stakeholder-engaged study that sought to identify factors influencing capacity and readiness to implement PSE interventions within food pantries.

## Methods

**Fig. 1.**
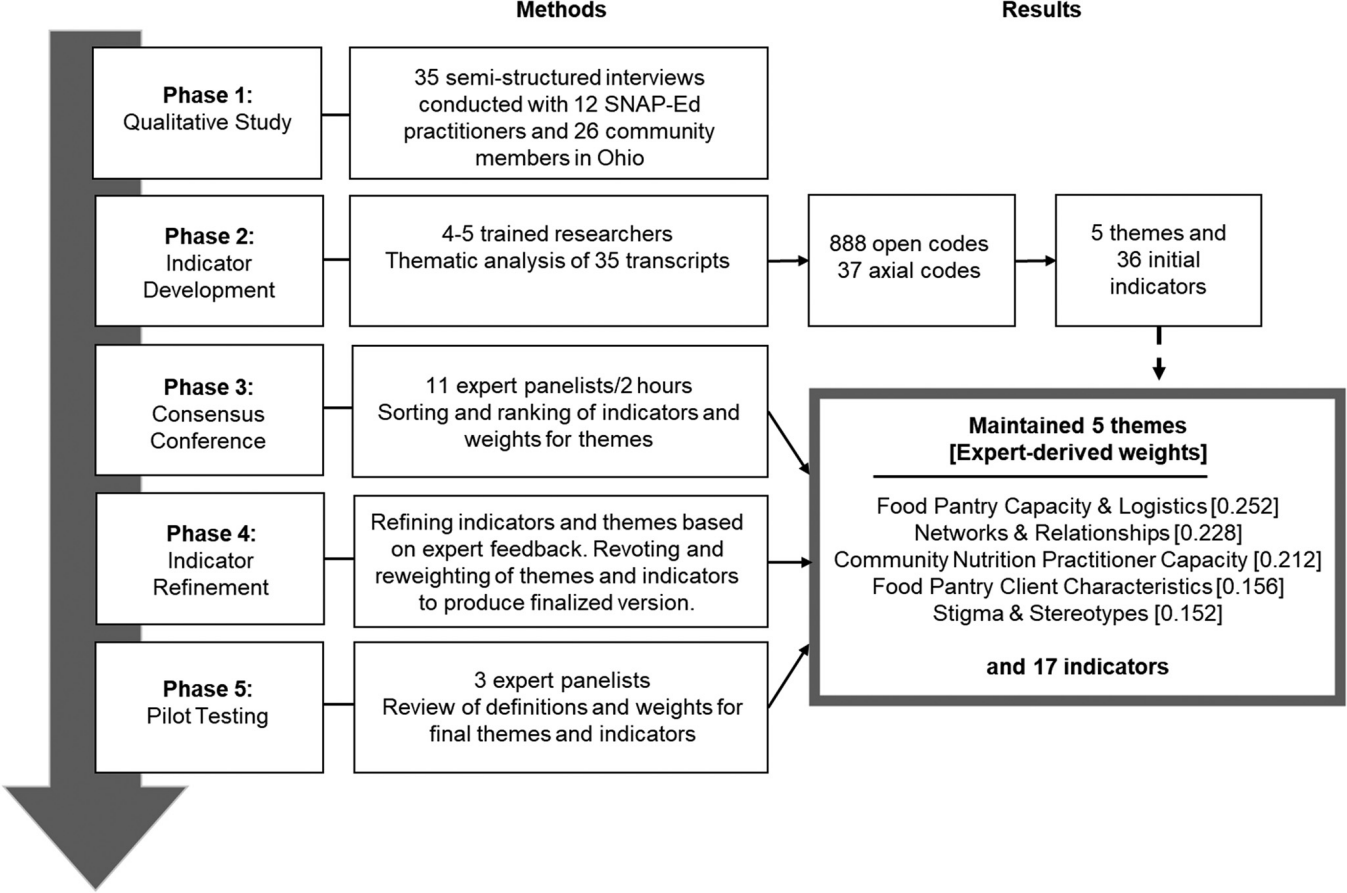
Multi-phase research approach to identify factors influencing implementation of healthy eating PSE interventions in food pantry settings based on perspectives of diverse stakeholders.

This multi-phase study was conducted through a partnership between public health researchers and the SNAP-Ed implementing agency in Ohio, The Ohio State University (OSU) Extension (see Fig. 1). OSU Extension SNAP-Ed is the only implementing agency in Ohio, and as such its jurisdiction is the entire state of Ohio. While Ohio SNAP-Ed does not provide funding directly for the pantry operations, food purchases and pantry staffing, they are able to teach SNAP-Ed classes in food pantries and support PSE interventions at food pantries. Our aim was to gather information from four stakeholder groups who offered different perspectives about PSE interventions in food pantry settings, including: (1) county-level SNAP-Ed practitioners, (2) SNAP eligible residents community residents with experience receiving SNAP benefits, (3) practitioners with experience managing food pantry programming and (4) researchers with expertise in food pantry-based research. The study was approved by Case Western Reserve University Institutional Review Board (IRB). The consolidated criteria for reporting qualitative research (COREQ) was used to report methods and results in this study (see Supplementary Table S1)^(20)^.

### Phase 1: Qualitative study

Phase 1 included qualitative data collection with county-level SNAP-Ed Program Assistants (referred to as ‘practitioners’ moving forward) (*N* 12) and community members residing within the target counties (*N* 26) (referred to as ‘residents’ moving forward). Participants were recruited from five urban and four rural counties in Ohio that were purposively sampled to include counties that had different Rural–Urban Commuting Area (RUCA) codes and obesity rates. Six of the twelve practitioners were interviewed in pairs because they worked within the same county, thus three transcripts from six practitioners and six transcripts from the remaining six (totalling nine interview transcripts for practitioners) and twenty-six transcripts for residents made a total of thirty-five transcripts. Prior to the study, practitioners had a working relationship with the researchers through an organisational partnership between the research centre and Ohio SNAP-Ed. Email invitations were sent by Ohio SNAP-Ed leadership to county-level practitioners to assess their interest in study participation. Thirteen practitioners expressed interest in the study by contacting research staff by email or phone and consented to participate in the study. Of those that expressed interest, twelve practitioners were interviewed, and one practitioner did not respond to researcher's emails to schedule their interview. SNAP eligible community residents were recruited through flyers posted at local food pantries, public housing facilities, homeless shelters, schools, public assistance program offices (i.e. Special Supplemental Nutrition Program for Women, Infants and Children (WIC), Head Start) and social media pages. Thirty-seven of the community residents consented to participate in the study, among which 70⋅3 % completed the interviews. The remaining 29⋅7 % either did not respond to follow-up emails/phone calls to schedule interviews or did not show up for their interview. As part of the consent, participants received information about the goals of the research and had the opportunity to ask researchers questions before consenting to participate in the study. Informed consent was obtained over the phone for all participants as COVID-19 mitigation strategies were in place during the study timeframe. All interview guides were designed by researchers (see Supplementary File S1 and S2). Both interview guides included questions to learn about the community and pantry capacity and support needed to carry out and sustain PSE interventions at food pantries. Interview guide for practitioners also included questions on previous experiences planning and implementing PSE interventions in food pantry settings. Prior to the data collection interviews, the researchers and three Ohio SNAP-Ed PSE specialists conducted three mock/pilot interviews. Interview guides were revised based on feedback from the mock interviews.

In-depth interviews were conducted virtually between December 2020 and May 2021, at participants’ convenience and in a private, virtual space. Each interview had two research team members who served as a facilitator or notetaker (OY, JS and LO). All researchers had extensive experience and training in qualitative research methods including data collection and analysis. Additional training was provided prior to data collection. At the beginning of each interview, researchers introduced themselves sharing their names and roles within the organisation and the interview. The practitioner interviews lasted approximately 1⋅5–2 h. After each interview, the interviewer and notetaker met to debrief. Demographic information was not collected from practitioners to maintain confidentiality. The resident interviews lasted between 20 and 60 min and the questions aimed to gather feedback on interests as well as current and future needs for interventions in food pantries. At the end of the resident interviews, demographic information was collected. All interviews were audio recorded and transcribed verbatim by a third-party transcriptionist. The transcripts (*N* 35) were reconciled for accuracy before data analysis.

### Phase 2: Indicator development

Phase 2 consisted of three stages: open coding, axial and higher-level coding, and indicator development. Four researchers adapted an iterative process based on a grounded theory approach to open code^(21)^ (inductive and deductive) all transcripts using Nvivo 12. We combined all transcripts from both practitioners and residents because the interview guides were developed around the same framework. All transcripts were summarised line by line into *open codes*, which reflected the actual words from participants. At the beginning of the coding process, all four researchers coded the same transcript to ensure consistency^(22)^. After coding three transcripts together and achieving agreements, transcripts were split among researchers to code individually.

Following the open coding process, all open codes were then grouped into *axial codes* based on the similarities in the emerging concepts. All axial codes were then grouped into *higher-level codes* based on existing theories related to community readiness and organisational capacity for the implementation of healthy PSE interventions^(17,23)^, as well as the emergent themes from our analysis. Researchers met weekly to reconcile axial codes and higher-level codes. Areas of disagreement were discussed during weekly meetings and consensus was reached following discussions.

The final stage of the data analysis process involved indicator development. Five researchers created a series of related questions, herein referred to as ‘indicators’ that reflected the main idea in each axial code. The number of indicators for each higher-level code varied based on the number and depth of embedded axial codes^(22)^. These indicators represent key factors associated with community readiness and capacity for implementing PSE interventions within food pantries. Overall, our data yielded thirty-seven axial codes, thirty-six indicators and five higher-level codes (themes) related to readiness for implementing healthy eating PSE interventions in food pantries. While some codes and themes were more prevalent in either the resident or practitioner transcripts, overall, there was significant overlap that reinforced the results from this study.

### Phase 3: Consensus conference

During Phase 3, a panel of experts met to assess the relevance and importance of the higher-level codes and indicators, as well as to group indicators into the themes that were provided. Of the twenty-one expert panelists that were invited through email across the state of Ohio, 11 (52⋅3 %) participated in a virtual meeting. The panelists were invited based on their knowledge and experience with food pantries, community nutrition practices and/or low-income populations utilising food pantries.

During the virtual meetings, two activities were performed using a free online application for virtual collaboration. All expert panelists were given the themes and their definitions developed in Phase 2 prior to the meeting and for use during the activities. During the first activity, the expert panelists were grouped in pairs or triads based on similarities in their roles and settings of work. They collaboratively sorted indicator questions into the themes generated from Phase 2 (Indicator Development). The expert panelists then worked together in the same groups to rank the indicators within each theme by order of importance for successful implementation of food pantry PSE interventions.

For the second activity, ten of the eleven expert panelists worked independently to assign weights to each of the food pantry themes on a scale of 0–25. Each expert distributed their 25 points across the five themes on their designated collaborative online board, assigning more points to the themes they felt were most important and relevant for successful implementation of food pantry PSE interventions. From these activities, a new indicator (Indicator 2.1) was suggested based on conversation with experts. After the meeting, the research team assigned a score to the top three indicators within each theme and derived indicator weights following the approach adopted by Lee *et al.*^(24)^.

### Phases 4 and 5: Indicator refinement and pilot testing

After the consensus conference, the research team refined the themes and indicators embedded in each theme based on expert feedback (see Fig. 1, Phase 4). The refinement process ultimately included the removal of redundant indicator questions, addition of a new indicator question (Indicator 2.1), and the overall clarification of theme titles and indicator language.

Once these changes were made, the same group of experts was asked via email to reassign weights to the refined themes and re-rank the indicators within two themes (repeating Phase 3). Given that the refined theme descriptions were not significantly different from the ones that were used in the consensus conference, researchers carried forward the theme weights from the two experts who did not participate in the re-voting exercise. The results of this re-voting process yielded the final weights for the five themes and seventeen indicator questions, which were then pilot tested with researchers with expertise in food pantry-based research (*N* 3) to assess content and overall validity of the assessment tool (see Fig. 1, Phase 5). The research team revised one indicator based on feedback from these stakeholders.

## Results

Overall, a total of thirty-eight participants were interviewed: twelve practitioners and twenty-six residents. Most residents were female (76⋅9 %), over the age of 50 (50 %) and resided in an urban county (69⋅2 %). Regarding race and ethnicity, 57⋅7 % of the residents identified as White, 23⋅1 % identified as Black or African American, 3⋅8 % identified as Hispanic or Latino and 15⋅4 % identified as Multiracial or some other race. All residents had experience (past or present) with SNAP, with 88⋅5 % actively using the program. Practitioners (*n* 12) interviewed had an average experience with SNAP-Ed of 7⋅4 years, with an overall range of 1⋅5–20 years. Participants were assigned a unique identification number (P1–P4 for practitioners; R1–R11 for residents). For each quote identified in the results, they are labelled with the identification number of the participants associated with it.

Five themes emerged from our qualitative analysis process. Themes, with their expert-derived weights, are: (1) Food Pantry Capacity and Logistics [0⋅252], (2) Networks and Relationships [0⋅228], (3) Community Nutrition Practitioner Capacity [0⋅212], (4) Food Pantry User Characteristics [0⋅156] and (5) Stigma and Stereotypes [0⋅152]. Overall, seventeen indicators were identified to operationalise these themes.

### Food Pantry Capacity and Logistics

The Food Pantry Capacity and Logistics theme encompasses food pantry operations that influence the implementation of PSE interventions. This emerged as the highest ranked theme with an expert-derived weight of 0⋅252. These operations include hours of service, distribution models, storage capacity, food sourcing, staffing, etc. Table 1 shows the four final indicators listed in order of priority.

**Table 1.** Themes and indicators and their perceived importance (weights) to the implementation of healthy eating PSE interventions in food pantries

Theme	Theme weight	Indicator number	Indicator question	Indicator weight
Food Pantry Capacity and Logistics	0⋅252	1.1	Storage capacity for healthy, perishable foods	0⋅333
		1.2	Recruitment and training of food pantry staff and volunteers	0⋅278
		1.3	Consistent and reliable healthy food sourcing systems	0⋅222
		1.4	Choice within food pantries	0⋅167
Networks and Relationships	0⋅228	2.1	Relationships with food sourcing systems	0⋅478
		2.2	Community awareness of available healthy food options	0⋅261
		2.3	Community stakeholder connections	0⋅261
Community Nutrition Practitioner Capacity	0⋅212	3.1	Prioritising food pantry PSE interventions within community nutrition organisations	0⋅432
		3.2	Resources to support food pantry PSE interventions	0⋅295
		3.3	Regular engagement with food pantries	0⋅273
Food Pantry Client Characteristics	0⋅156	4.1	Culinary skills among food pantry users	0⋅412
		4.2	Prioritising food taste and preference within food pantry system	0⋅294
		4.3	Household food habits and choices	0⋅176
		4.4	Food pantry clients’ willingness to choose healthy food options at pantries	0⋅118
Stigma and Stereotypes	0⋅152	5.1	Stigma and judgement associated with food pantry usage	0⋅409
		5.2	Perceptions about the food pantry operations and personnel	0⋅409
		5.3	Perceptions about the quality of fresh and healthy food options available in food pantries	0⋅182

#### Indicator 1.1: Storage capacity for healthy, perishable foods

Since food pantries operate in a variety of spaces, their internal capacity to store and distribute food products can vary. Residents and practitioners expressed concerns regarding a food pantry's internal capacity to store fresh, perishable foods. For example, one resident discussed that the available apples were sometimes rotten because the pantry lacked the necessary storage and/or ‘cooling system’ needed for perishable foods (R1). Another resident discussed that, due to the poor condition of fresh fruits offered, they prefer selecting ‘canned pineapples’ over fresh pineapples (R2).

#### Indicator 1.2: Recruitment and training of food pantry staff and volunteers

Participants discussed that a pantry's ability to recruit, train and retain staff and volunteers is essential for maintaining operations, as well as initiating, implementing and sustaining PSE interventions. Practitioners discussed that the limited availability and high turnover of pantry staff and volunteers created a difficult environment for initiating contact and maintaining relationships with pantry personnel that are necessary for implementing PSE interventions. Pantry personnel were perceived as ‘really busy’, ‘overwhelmed’ and ‘hard to pin-down’ (P1). Practitioners identified these staffing constraints as a major barrier and expressed that pantry personnel had limited time to commit to PSE intervention work.

#### Indicator 1.3: Consistent and reliable healthy food sourcing systems

Residents and practitioners stressed the importance of having reliable sourcing systems that provide fresh and healthy foods to food pantries. Both residents and practitioners felt that food donated to pantries were usually not nutritious, ultimately posing a challenge for food pantry users to eat healthy as the following quote highlights.

‘I don't think that some of the stores that donate to the food pantries always donate necessarily healthy items, and not necessarily items that would even make any type of meal. So … when you go to a food pantry, [it's] hard to make a healthy meal … ’ (R3).

One practitioner indicated that some food pantries provide specific nutritional guidelines for the food they accept from donors. When pantries have funds to purchase food, one practitioner reported that some will make sure they order food items that ‘cover all the five food groups’ (P3).

#### Indicator 1.4: Choice within food pantries

Residents expressed the desire to have more food options and the ability to choose items within their pantry. One resident discussed that they would prefer to choose a different kind of meat at the pantry because they are ‘chickened out’ from only having one meat option available (R2). Practitioners also echoed the importance of choice, citing various levels of client choice pantry models that they observed or envisioned within collaborating food pantries. For example, one practitioner expressed interest in a choice model in which food pantry users could ‘go through and pick’ items they want, like shopping at a grocery store (P4).

### Networks and Relationships

The Networks and Relationships theme refers to partnering with key personnel, groups or organisations that aid practitioners in the implementation of food pantry PSE interventions. This emerged as the second highest ranked theme, with an expert-derived weight of 0⋅228. The three final indicators prioritised by experts, listed in order of perceived importance, can be found in Table 1.

#### Indicator 2.1: Relationships with food sourcing systems

This indicator emerged from the consensus modelling as the expert panelists discussed the critical role of food sourcing systems in the variety, quantity and quality of food at food pantries. By forming stronger relationships with a wide range of sourcing organisations, including food banks and local grocery stores, pantries can provide a variety of quality food options to their users. According to the experts, failure to establish connections with food sourcing organisations, such as food banks, could limit client food selection at food pantries and hinder the planning and implementation of healthy food PSE interventions.

#### Indicator 2.2: Community awareness of available healthy food options

Our interviews revealed that the level in which food pantries coordinate with community leaders to promote healthy food inventory varies significantly across pantries and communities. One resident discussed that a leader in their community ‘sends out group emails to many community members asking for input or resources’ for a food pantry in the area (P3). In this instance, the participant described the community leader as an ‘advocate’ who was instrumental in the formation and communication platform of a school pantry. Similarly, participants discussed that additional awareness-building techniques for PSE interventions can involve spreading information by word-of-mouth, flyers and social media. Regardless of methodology, connecting to community messengers was viewed as producing effective outreach results.

#### Indicator 2.3: Community stakeholder connections

Practitioners discussed they must spend significant time fostering relationships with key community stakeholders, community coalitions and professional networks. Practitioners cited a wide range of partners within the community that could support the implementation of PSE interventions at food pantries, including churches and government offices.

‘I do have some connections … I know that my church has a food pantry that they're working with, so just connecting with churches, [state government agencies] might have some connections with different food pantries, as well as hospitals’ (P4).

Practitioners discussed that these long-standing relationships and potential partners within their community are vital to the funding, maintenance and sustainability of PSE interventions in food pantries.

### Community Nutrition Practitioner Capacity

The Community Nutrition Practitioner Capacity theme refers to the skills, resources and capacity of practitioners from SNAP-Ed and other community nutrition and public health organisations to support the implementation of food pantry PSE interventions. This is ranked third among the themes with an expert-derived weight of 0⋅212. Table 1 shows the three final indicators prioritised by experts listed in order of perceived importance.

#### Indicator 3.1: Prioritising food pantry PSE interventions within community nutrition organisations

During the interviews, practitioners expressed that it was important for community nutrition organisations, such as SNAP-Ed, to prioritise PSE interventions. This could include having dedicated staff committed to planning and implementing PSE interventions in food pantries. Practitioners discussed how they feel supported by dedicated regional staff who provide PSE guidance. The existence of these regional staff members, referred to as ‘PSE Specialists’ demonstrates structural support within the organisation that goes beyond setting PSE interventions requirements. Despite this built-in support, some practitioners discussed that incorporating PSE interventions into their schedule can pose a challenge with other organisational priorities (i.e. direct education). One participant expressed challenges establishing this work balance by stating that ‘a lot of it [PSE interventions] feels like extra credit’ and can be deprioritised in relation to other job requirements (P1).

#### Indicator 3.2: Resources to support food pantry PSE interventions

Organisation and program resources (i.e. funding, time, staff) to support PSE interventions were identified by practitioners as a key factor for the successful planning and implementation of food pantry interventions. During the interviews, practitioners expressed concern about the limited funds available ‘to support some of the things that [they] would like to do’ within food pantries (P2). To expand on this thought, the practitioner described their experience with limited funding for these activities:

‘I work with some organizations … who have a lot of funds that they can put towards PSEs and that's the main reason that I think our county has had a lot of success with PSEs, and so in areas where that's not available, there's no financial resources and there's not somebody kind of driving those PSEs and getting them started, it's much more challenging … ’ (P2).

#### Indicator 3.3: Regular engagement with food pantries

Interviews revealed that practitioners had different levels of engagement with food pantries and ultimately set different goals for PSE interventions. In most instances, practitioners provided nutrition education at food pantries. Other forms of engagement included surveying pantry clients about healthy food access, supporting food labelling and sorting processes, training volunteers at food pantries and facilitating partnerships between pantries and other community organisations. For example, one practitioner was ‘able to do a survey with some clients at the food pantry as far as issues with where they bought their fruits and vegetables and if they had difficulty getting those’ (P2). Ultimately, practitioners discussed that it was important to regularly engage with food pantries in their service area to understand pantry operations, (continually) assess their capacity to offer healthy eating PSE interventions and uncover potential areas for future work.

### Food Pantry Client Characteristics

The Food Pantry Client Characteristics theme refers to residents’ knowledge of healthy eating, culinary skills and food habits. This theme was ranked fourth among the five themes with an expert-derived weight of 0⋅156. Overall, this theme captures specific factors that may limit or enhance food pantry clients’ ability to prepare and eat healthy foods, including food prep knowledge and familial food norms (i.e. food preferences). The four final indicators prioritised by experts and listed in order of perceived importance are shown in Table 1.

#### Indicator 4.1: Culinary skills among food pantry users

Both residents and practitioners expressed that healthy eating PSE interventions in food pantries may require those who use pantries to have culinary skills to prepare meals with fresh and healthy produce. One resident discussed that ‘you can get all this stuff [fresh and healthy produce] from the food pantry and not have a clue what to do with it’ (R1). This participant reiterated the sentiment, stating:

‘There's two churches that have food pantries where you come get food, … but the problem with it is, there's no education with it. You just get a free meal, and a lot of things you get at a food pantry, people have no idea what to do with’ (R1).

To address this concern, residents identified potential healthy eating activities they would like to see, such as providing recipes that aligned with their pantry's produce. One resident specifically stated, ‘it'd be awesome if they [food pantries] had activities there while you're picking up your items on how to cook certain items, or what to do with them in a recipe’ (R4).

#### Indicator 4.2: Prioritising food taste and preference within food pantry system

During the interviews, residents and practitioners expressed the desire for pantries to consider food options that align with pantry clients’ cultures, taste preferences and dietary restrictions. One resident indicated that food pantries must ‘find out about what kind of food each culture eats, instead of just giving [food pantry clients] anything, assuming that they eat anything and everything’ (R5). Other residents interviewed indicated that their food preferences were based on personal choice and health-related reasons. Aligning pantry resources with pantry clients’ food preference could reduce food waste and increase food consumption, as highlighted by one resident (R12).

#### Indicator 4.3: Household food habits and choices

Food habits and choices were identified as important determinants of eating patterns among those who visit pantries. For most participants, this concept was validated when stating that their healthy eating choices were heavily influenced by their upbringing. One resident explained that they eat healthy foods because they ‘always had a good home-cooked meal’ when they were growing up and ‘still make[s] *their* own good food’ (R6). This resident discussed that training their seven children to eat healthy has resulted in healthy eating among their grandchildren:

‘I've got seven kids. They're grown and they're doing the best they can for my three grandkids, and we do really well with teaching them and making sure they eat and everything and doing the right thing’ (R6).

Overall, both practitioners and residents stressed that childhood exposure to healthy eating within households could result in lifelong healthy eating behaviours.

#### Indicator 4.4: Food pantry clients’ willingness to choose healthy food options at pantries

Both residents and practitioners discussed that people who utilise food pantries, and who periodically face household food insecurity, are less likely to prioritise healthy eating due to various competing priorities. One resident explained that whenever they visited the food pantry, their priority was to make sure they ‘had food and it tasted good, as opposed to whether it was fruits, vegetables, and whole grains’ (R7). Practitioners rationalised this decision making because they see people visiting pantries are often ‘just trying to get some food in their kids’ mouths and get them to bed’ as opposed to telling their kids that ‘half of [their] plate should be fruits and vegetables’ (P4). To promote healthy eating behaviours, one practitioner observed that some food pantries have adopted various programming options that promote and reward healthy food selection at their pantries.

### Stigma and Stereotypes

The Stigma and Stereotypes theme refers to the internal and external judgement associated with using a food pantry. This includes how residents perceive food pantry operations, personnel, users and the quality of food available. This theme was ranked fifth, with an expert-derived weight of 0⋅152. Table 1 shows the three final indicators prioritised by experts and listed in order of perceived importance.

#### Indicator 5.1: Stigma and judgement associated with food pantry usage

Stigma was identified by residents as a limiting factor to using food pantries in their communities. Some residents described that food pantry usage is sometimes associated with financial insecurity and/or ‘feeling like a failure’ (R8). One resident discussed that this stigmatising association prevents families in need from using food pantries because ‘they don't want people to know that they're struggling’ (R9). This is evident in conversations with another resident who was reluctant to initially use food pantry assistance when their family was facing financial difficulties:

‘People came and brought a box to our house … We go, ‘Oh no (x3). We can fend for ourselves. Somebody else is more needy. We don't need that,’ but the lady that brought

the box says, ‘That's good, but somebody wanted you to have this. You've got to be humble enough to accept it’ (R10)

#### Indicator 5.2: Perceptions about the food pantry operations and personnel

Residents expressed that community perceptions about food pantry personnel and operations were important to consider when implementing healthy eating interventions. There was some discussion around (mis)trust in personnel who distribute produce to those in need, with one resident stating that they ‘went to a pantry where they had stuff stacked on the side, … for somebody else … ’ and personnel were ‘not giving out the fruits and vegetables the way they're supposed to’ (R5). Practitioners reiterated that successful PSE interventions at the food pantry will require staff and volunteers to treat all visitors equitably and foster an environment of trust.

#### Indicator 5.3: Perceptions about the quality of fresh and healthy food options available in food pantries

The community's knowledge of food pantry operations, including the type and quality of food available at food pantries, can affect the implementation of PSE interventions at food pantries. Residents revealed that many people ‘look down on food pantries’ (R11) because they assume that the food provided at the pantry is of poor nutritional quality. The same resident indicated that people in their community ‘steer away from food pantries because they don't think they'll get good stuff’ (R11). The perceptions of food quality at pantries can negatively affect pantry utilisation, even among households in need. Changing the perceptions of the food pantry experience and quality of food available will be a necessary step that practitioners need to take to increase intervention buy-in.

## Discussion

The themes and indicators developed through this study represent the range of factors that influence the capacity and readiness to implement healthy eating PSE interventions in food pantries. Our findings reveal that successful and sustained PSE change will require a tailored approach that considers pantry and community-level factors as well as partnerships in the development and execution of PSE intervention in these settings.

Food pantries are unique settings for implementing PSE interventions due to differences in their operations, size, partner organisations, funding sources and governing structure. Additionally, we find that the variation in selection and quality of food available at food pantries limit what every food pantry could offer. This heterogeneity within pantries and other community/system-level dynamics requires tailored food pantry interventions. Generalised healthy food pantry interventions do not always consider the nuances of the food pantry's sourcing, capacity and limitations. Additionally, a generalised framework fails to adapt to situations in which dramatic change (e.g., COVID-19 pandemic) greatly impacts food pantry capacity and operations. Research shows during the earlier periods of the pandemic, emergency food programs adapted their approach to networking, partnering and food distribution in order to meet the increased demands throughout pantries and the food system as a whole^(25–27)^. These adaptations differed from pantry to pantry. Therefore, a tailored intervention is necessary to sustain PSE success, not only during normal operations at the pantry, but also in times of crisis.

Interventions at food pantries have predominantly focused on increasing availability of fresh and healthy foods within the food pantry setting^(8,10–12)^. In most studies/interventions, access to fresh and healthy foods has been discussed within a geographic or financial lens^(28)^. Most of these interventions, however, do not fully consider the sociopolitical context influencing healthy eating patterns among people using food pantries. Our results reveal that stigma, stereotypes and community perceptions are significant barriers that delay access to healthy eating interventions at food pantries. As highlighted by indicators 5.1–5.3, these factors can encompass how residents perceive food pantry users, food pantry staff and the quality of food offered at pantries. Although researchers have investigated and identified stigma as a barrier to using food pantries^(29–31)^, this area has received less attention from interventionists because of its complexity and context-specific nature. Our results support these earlier findings and emphasise the need for food pantry interventions to target all potential community-specific barriers to access, including stigmatising perceptions surrounding pantry usage^(31,32)^.

Our results further reveal that sustainable PSE interventions at food pantries will require coordinated efforts, time and resources from a variety of stakeholders, such as food pantry personnel, donor organisations community nutrition organisations such as SNAP-Ed, and other public health institutions. The practitioners in our study alluded to issues regarding continuity of interventions due to lack of buy-in from partners and pantries inability to secure long-term funding and support. Current literature on food pantry interventions shows that most interventions are pilot studies, grant-funded and/or short-lived reviews^(8,9,12,33)^. The short lifespan of these interventions results in diminished long-term impacts on healthy eating. Our findings suggest that prioritisation of PSE interventions within food pantry settings, combined with strong, committed relationships among key stakeholders, such as SNAP-Ed, is important for the long-term success of these efforts.

Our findings support the need for a comprehensive planning tool that allows for the collaboration of diverse stakeholders and recognition of various individual- and community-level factors that influence healthy eating in food pantries. The themes and indicators produced from this study have been translated into an online assessment tool^(34)^ called The PSE Readiness Assessment and Decision Instrument (PSE READI). Informed by our data, this tool helps public health practitioners and their partners assess the capacity and readiness of their communities for tailored healthy eating PSE interventions in food pantries. Unlike current existing assessments that target some aspects of the pantry environment^(13)^, this tool provides comprehensive assessment of pantries’ readiness to implement PSE interventions. Upon completion, the tool produces community-tailored, next step recommendations that can guide practitioners and partners as they aim to conduct impactful and long-lasting community interventions for healthy eating.

We note that collecting this data during the COVID-19 pandemic may have influenced some of the findings from this study. Changes in pantry operations during the pandemic, increased clientele, and the limited availability of staff and volunteers during this time may have influenced residents’ and practitioners experience at food pantries and PSE interventions within these settings.

## Conclusion

Food pantries provide free food to households, including those receiving SNAP, thereby buffering food insecurity in communities. Given their vital positioning within the U.S. food system, the national SNAP-Ed program has identified food pantries as a targeted setting for implementing PSE interventions that promote healthy eating. In the present study, we partnered with Ohio SNAP-Ed to identify factors influencing capacity and readiness to implement PSE interventions within food pantries. Overall, we find that food pantries are unique settings for implementing healthy eating PSE interventions requiring tailored approaches to implementation based on local readiness and capacity. The themes and indicators identified through this study offer guidance for selecting and prioritising this type of tailored approach in diverse food pantry settings.

## Supplementary material

For supplementary material accompanying this paper visit https://doi.org/10.1017/jns.2023.64. click here to view supplementary material
