# Supplementary material for: Pantry clients and Supplemental Nutrition Assistance Program-Education practitioners’ perspectives on factors influencing healthy eating policy, system and environmental interventions in food pantries
Source: J Nutr Sci. 2023 Jul 19;12:e81. doi: 10.1017/jns.2023.64 (PMC10388411; doi:10.1017/jns.2023.64)
Supplement: Supplementary file 1 [file S2048679023000642sup001.docx]

Supplementary Table 1: Consolidated criteria for reporting qualitative studies (COREQ): 32-item checklist

| Pantry Clients and SNAP-Ed Practitioners' Perspectives on Factors Influencing Healthy Eating PSE Interventions in Food Pantries | | | |
| --- | --- | --- | --- |
| No | **Item** | **Guide questions/description** | **Manuscript page number** |
| Domain 1: Research team and reflexivity |  |  |  |
| Personal Characteristics |  |  |  |
| 1. | Interviewer/facilitator | Which author/s conducted the interview or focus group? | Page 7 |
| 2. | Credentials | What were the researcher's credentials? *E.g. PhD, MD* | Title page |
| 3. | Occupation | What was their occupation at the time of the study? | We have included our affiliated organizations |
| 4. | Gender | Was the researcher male or female? | We have not provided this information because we do not see how that impacts our study. |
| 5. | Experience and training | What experience or training did the researcher have? | Page 7 |
| Relationship with participants |  |  |  |
| 6. | Relationship established | Was a relationship established prior to study commencement? | Page 6 |
| 7. | Participant knowledge of the interviewer | What did the participants know about the researcher? e*.g. personal goals, reasons for doing the research* | Page 6 |
| 8. | Interviewer characteristics | What characteristics were reported about the interviewer/facilitator? e.g. *Bias, assumptions, reasons and interests in the research topic* | These were not reported. |
| Domain 2: study design |  |  |  |
| Theoretical framework |  |  |  |
| 9. | Methodological orientation and Theory | What methodological orientation was stated to underpin the study? *e.g. grounded theory, discourse analysis, ethnography, phenomenology, content analysis* | Page 8 |
| Participant selection |  |  |  |
| 10. | Sampling | How were participants selected? *e.g. purposive, convenience, consecutive, snowball* | Page 6 |
| 11. | Method of approach | How were participants approached? e*.g. face-to-face, telephone, mail, email* | Page 6 & 9 |
| 12. | Sample size | How many participants were in the study? | Page 5 & 9 |
| 13. | Non-participation | How many people refused to participate or dropped out? Reasons? | Page 6 |
| Setting |  |  |  |
| 14. | Setting of data collection | Where was the data collected? e*.g. home, clinic, workplace* | Page 7 |
| 15. | Presence of non-participants | Was anyone else present besides the participants and researchers? | Page 7 |
| 16. | Description of sample | What are the important characteristics of the sample? *e.g. demographic data, date* | Page 12 |
| Data collection |  |  |  |
| 17. | Interview guide | Were questions, prompts, guides provided by the authors? Was it pilot tested? | Page 7 |
| 18. | Repeat interviews | Were repeat interviews carried out? If yes, how many? | N/A |
| 19. | Audio/visual recording | Did the research use audio or visual recording to collect the data? | Page 8 |
| 20. | Field notes | Were field notes made during and/or after the interview or focus group? | Page 7 |
| 21. | Duration | What was the duration of the interviews or focus group? | Page 7-8 |
| 22. | Data saturation | Was data saturation discussed? | No- We ended data collection once we reached our recruitment target |
| 23. | Transcripts returned | Were transcripts returned to participants for comment and/or correction? | No- Through the consensus modeling exercise, we received feedback on the findings and results. |
| Domain 3: analysis and findings |  |  |  |
| Data analysis |  |  |  |
| 24. | Number of data coders | How many data coders coded the data? | Page 8 |
| 25. | Description of the coding tree | Did authors provide a description of the coding tree? | Page 8-9 |
| 26. | Derivation of themes | Were themes identified in advance or derived from the data? | Page 8-11 |
| 27. | Software | What software, if applicable, was used to manage the data? | Page 8 |
| 28. | Participant checking | Did participants provide feedback on the findings? | No |
| Reporting |  |  |  |
| 29. | Quotations presented | Were participant quotations presented to illustrate the themes / findings? Was each quotation identified? e*.g. participant number* | Page 13-26 |
| 30. | Data and findings consistent | Was there consistency between the data presented and the findings? | Page 12-26 |
| 31. | Clarity of major themes | Were major themes clearly presented in the findings? | Page 13-24 |
| 32. | Clarity of minor themes | Is there a description of diverse cases or discussion of minor themes? | Page 13-26 |

Supplementary File 1: Interview guide questions for Community Residents focused on Food Pantry PSE Interventions

1. **Getting to Know the Community**
2. Can you describe your community to us?
   1. What is the name of your community?
   2. Where is it located in the county?
   3. What are the things that make your community different than other communities in your county? (e.g., interesting landmarks, size, age/race/gender of people living there, access to resources)
   4. What are some of the interests in your community related to healthy eating?
   5. What are some of the problems or concerns in your community related to healthy eating?
      1. Are there specific groups in your community with greater problems or concerns related to healthy eating?
   6. How is COVID-19 influencing healthy eating in your community? Is anything making it easier or harder to eat healthy during this time?
3. What do you think people in your community need in terms of healthy eating activities? As a reminder, examples of these include – **[Refer to list on Handout 1]**
   1. Why do you think these types of activities are wanted or needed in your community?
   2. Are any of these efforts happening now in your community? If yes, which ones? (i.e., can probe about SNAP-Ed programming if relevant)
   3. How are needs for healthy eating activities in your community changing because of COVID-19?

[Research Team]*: The next question is the first of 3 questions that I’m going to ask you to rate something on a scale of 1-10.*

1. On a scale from 1-10 with 1 being “not at all” to 10 being “a great deal”, to what degree do *healthy eating* activities fit into the culture of your community? When I say culture, I mean how things are normally done or what people believe in. **[Refer to list on Handout 1]**

Can you tell me why you selected a ___?

How do the culture or values of the community make it **easier OR harder** to eat healthy?

1. **Specific PSE Activities**
2. On a scale from 1-10 with 1 being “no interest at all” to 10 being “a great deal of interest”, how interested are you, in terms of **yourself and your family**, in having healthy eating activities in your community that focus on food pantries?
   1. Can you tell me why you selected a ___?
   2. How has COVID-19 influenced your thoughts about healthy eating activities in your community focused on food pantries?
3. On a scale from 1-10 with 1 being “no interest at all” to 10 being “a great deal of interest”, how interested do you think the **larger community** is in having healthy eating activities in your neighborhood that focus on food pantries?
   1. Can you tell me why you selected a ___?
   2. What are some of the **reasons** for starting healthy eating activities in food pantries in your community? (e.g., have people such as neighbors or members of a coalition in the community said they were interested in this, are there any examples of successes related to this in your community)
   3. What are some of the **problems** in starting healthy eating activities in food pantries in your community? (e.g., lack of support for these by key stakeholders like city council, social service agencies, community leaders; policies that make it difficult to create these types of changes)
4. What specific types of healthy eating activities in K-12 schools and food pantries would make the **biggest change** for you and others in your community? (e.g., would reach the most people, would result in the greatest benefits)
5. Do you have a recommendation for a specific person you know who receives or is eligible for SNAP that you think has a similar or different perspective than you that would be good for us to speak with? [If yes, remind participant about county eligibility, and ask the participant to share **our** study information out]
6. As a study we are trying to get more community residents to participate. Can you tell me how you were recruited to the study today?
7. What do you think is the more effective way to recruit residents?
8. **Demographics**

Research Team: *Before we finish today, we would like to gather a little information about who you are.*

1. What is your gender?
   1. Female
   2. Male
   3. Prefer not the answer
2. What is your age?
   1. 18 – 29 years
   2. 30 – 39 years
   3. 40 – 49 years
   4. 50 – 59 years
   5. 60 years or older
3. Do you currently have children under the age of 18 years living in your home?
   1. Yes
   2. No
4. Are you currently receiving SNAP benefits?
   1. Yes
   2. No
5. Are you currently receiving WIC benefits?
   1. Yes
   2. No
6. What is your race or ethnicity? Select all that apply.
   1. Black or African American
   2. White
   3. Hispanic or Latino
   4. Asian or Pacific Islander
   5. Native American
   6. Other, please describe: ______________________________
7. What is the name of your county? __________________________________

Supplementary File 2: Interview guide questions for SNAP-Ed Practitioners focused on Food Pantry PSE Interventions

1. **Getting to Know Participant**

[Research Team]: *As a reminder, the first set of questions are focused on you and your training and work experiences.*

1. How long have you worked at ______?
2. What motivated you to want to work in this position?
3. What did you do before working at ____?
4. Have you had any trainings that focus specifically on PSEs to prevent and reduce obesity trends? Please provide details for each training.

- ***Probe: these could be conference call opportunities or in-person trainings that OSU/SNAP-Ed hosts or supports***

| Training Name | Focus of Training | Any other relevant information |
| --- | --- | --- |
|  |  |  |
|  |  |  |
|  |  |  |
|  |  |  |

1. Do you have any prior experience related to Healthy Eating PSEs in Food Pantries

| Please describe each experience. | What role did you play in this experience? (i.e., leadership, participant, observer) |
| --- | --- |
|  |  |
|  |  |
|  |  |
|  |  |

1. Who have you or would you go to for information or support to plan and carry out the following PSE activities? It is ok to say “I don’t know.”

- ***Probe: this could be a partner within your professional network or “gatekeeper” in the community***
- ***[Interviewer Tip: Don’t go into too much detail. Looking to see how connected these people are- Do they know the key stakeholders and how well?]***

|  | **Key Contacts for Information** |  |
| --- | --- | --- |
| **PSE Intervention: Healthy Eating PSEs in Food Pantries** | **Organization/**  **Role** | **Why would you go to this person? Probes:**  **--knowledge**  **--support**  **--motivation**  **--general guidance**  **--trouble shooting** |
|  |  |  |
|  |  |  |
|  |  |  |

1. **Initiative Planning Processes**

[Research Team]: *The next questions are focused on the processes involved in your planning and carrying out of healthy eating PSE activities. From now on we’ll only be discussing these types of PSEs that are targeted on low-income populations in your county/community.*

| What types of healthy eating PSE activities focused on low-income populations are you currently involved with? | What most excites you about these activities? | What most concerns you about these activities? | Why were these initiative(s) selected?   - Did you model your program on existing activities? |
| --- | --- | --- | --- |
|  |  |  |  |
|  |  |  |  |
| c. |  |  |  |

1. What types of information (i.e., data, community meetings, surveys, recommendations from outside, stakeholder/coalition influence) do you use to prioritize healthy eating PSE activities for children and other residents in low-income populations in your community/county?

***[Interviewer Tip: In general, we are trying to figure out if people have a sense of where to go for information.]***

1. Where do you go to get the aforementioned information when you want to learn more about the following? It’s ok to say “I don’t know.” ***[Interviewer Tip: Ask about each question below as it appropriately applies. You do not need to ask them all if you don’t think they are applicable.]***
   1. Obesity trends or patterns in your community/county?
   2. Food insecurity or hunger trends or patterns in your community/county?
   3. Inequities (social, economics, ethnic, racial, etc.) related to obesity among residents in your community/county?
   4. Things that seem to be influencing obesity trends or patterns such as dietary behaviors among youth and adult residents in your community/county?
   5. The broader county/community context related to poverty, education levels, racial and ethnic diversity?
2. How do you evaluate your healthy eating PSE activities that target low-income populations in your community? Do you use any specific measures or outcomes? Why?
   1. In what ways, if at all, are the evaluation results being used to make changes in your current PSE activities or inform the start of new ones?
3. Now we’d like to get some more details about the food pantry PSEs you’re working on.

***[Interviewer tip: if they are not involved with food pantry PSEs, you can skip to question 11.]***

| About how many community stakeholders are aware of the PSE activities related to Healthy Eating PSEs in Food Pantries that you or your organization are working on? Would you say *none, a few, some, or most*? | Why do you say it’s _____? | How do community members learn about the current PSE activities related to Healthy Eating in Food Pantries you or your organization are working on? |
| --- | --- | --- |
| a. |  |  |
| b. |  |  |
| c. |  |  |

| Are there misconceptions or incorrect information among community members about the PSE activities related to Healthy Eating PSEs in Food Pantries that you or your organization are working on? If yes: What are these? | Do community members view current [name of healthy eating PSE in food pantries (e.g., your food pantry/food distribution project)] as successful?  *Probes: What do community members like about these activities?  What don’t they like? What are the perceived strengths and weaknesses of these activities?* | What are some of the obstacles for participating in [name of healthy eating PSE in food pantries (e.g., obstacles specifically related to COVID-19)]?  ***Among individuals*** *(e.g., hours not good, parents or other residents don’t want to participate, etc.)*  ***Among organizations*** *(e.g., food pantry is resistant to change)* |
| --- | --- | --- |

1. Are you planning to carry out any new PSE activities in your county/community?

| Please tell me about these activities (get information for all healthy eating PSEs). | Where are you in terms of carrying out the PSEs (i.e., currently planning, actively implementing, expanding, etc.)? | Who is involved in this work?   - Your organization - Community partners (i.e., government, community-based organizations, businesses, faith-based) - Coalitions - Other key stakeholders | Why was this PSE intervention selected?   - Community motivations and barriers - Personal motivations and barriers |
| --- | --- | --- | --- |
|  |  |  |  |
|  |  |  |  |
|  |  |  |  |

1. **Community Readiness**

[Research Team]: *We are now a little more than halfway through the interview. Would you like to get a drink of water, go to the bathroom, etc…?*

*The next questions are about your county/community* *and its readiness for supporting the carrying out of healthy eating policy, systems, and environmental activities. Again, we will be focusing on efforts to prevent and reduce obesity trends particularly among low-income populations. For this section, please think about ____ [name of community/county].*

1. Using a scale from 1-10, how much of a concern is obesity prevention among low-income populations in your community, with 1 being “not a concern at all” and 10 being “a very great concern”? **[See Handout 2** **for visual of scales.]**
2. Are there local entities that are more or less concerned about this (i.e., key leaders in the community, parents of children in low-income populations)?
3. Given COVID-19, how do you see concern for obesity changing over the next 12 months in your community?
4. Are there currently healthy eating PSE activities in [community] that **directly** address obesity (other than the healthy eating PSEs we have already discussed)?
   1. If **NO**, is anyone in [community] trying to get healthy eating PSE activities started to address obesity among low-income populations? Can you tell me about that?

***[Interviewer Tip: Once answered, skip to Section VI.]***

- 1. If **YES**, can you briefly describe each of these activities?
     1. How long have each of these activities been going on?

***[Interviewer Tip: Probe for each initiative/activity named.]***

1. **Organizational Readiness**

[Research Team]: *The next questions are about* ***your organization*** *and its readiness for supporting the carrying out of healthy eating policy, systems, and environmental activities. Again, the focus is on activities to prevent and reduce obesity trends particularly among children and adults in low-income populations. For this section, please think about [name of organization]*

1. Using a scale from 1-10, how much of a concern is obesity prevention among low-income populations to key leaders in your organization, with 1 being “not a concern at all” and 10 being “a very great concern”? **[See Handout 2** **for visual of scales.]**
   1. Can you tell me why you say it’s a _____?
   2. Is this changing due to COVID-19?
2. Among leadership in your organization, how much of a priority is addressing obesity among low-income populations through healthy eating PSEs activities with 1 being “not a priority at all” and 10 being “a very high priority”?
   1. Can you tell me why you say it’s a _____?
   2. Is this changing due to COVID-19?
3. How does leadership in your organization show support for healthy eating PSEs? For example, is it passive support or are they actively involved in such things as planning or participating in the PSE activities?
   1. If they are actively involved, how so?
   2. Are they involved in a committee or planning group?
   3. Do they speak out publicly to show support?
   4. Have they allocated resources for healthy eating PSE activities?
4. Does your organization have prior experience conducting healthy eating PSE activities to prevent or reduce obesity among children and other residents in low-income populations (other than the healthy eating PSEs we have already discussed)? If yes, please describe the following:
   1. Who were the people most supportive of these activities within your organization?
   2. Who were the people opposing these activities within your organization?
5. What other organizations (i.e., civic, faith-based, government, business) does your organization regularly partner with?
   1. What is your role in these partnerships?
   2. Are any of these partnerships helpful for carrying out healthy eating PSEs?
   3. Do you think there would be a need to form any new partnerships to carry out healthy eating PSEs?
6. What is the image of your organization in your county/community? Why?
   1. **Probe: Do people see your organization in a positive way? Or a negative way?**
   2. Among key leaders in the county/community?
   3. Among low-income populations in the county/community?
7. Using a scale from 1-10, with 1 being “an individual problem (e.g., lack of knowledge, skills, motivations)” to 10 being “a community problem (e.g., lack of access to healthy foods, too many unsafe parks)” how do **people in your organization** understand the problem of obesity among children and other residents in low-income populations in your county/community? [**See Handout 2** **for visual of scales.]**
   1. Can you tell me why you say it’s a _____?
8. In what ways, if any, does your organization actively seek feedback from the community stakeholders and other residents in low-income populations to guide your healthy eating activities?
9. On a scale from 1-10 [*with 1 being “not aware at all” and 10 being “a very high level of awareness”],* How aware is your organization of the demands of carrying out healthy eating PSEs to prevent or reduce obesity among children or other residents in low-income populations? *For instance, the demands related to relationship building, engagement with new partners, securing resources, working toward policy changes?* [**See Handout 2** **for visual of scales.]**
   1. Can you explain why you say it’s a _____?
10. **Specific PSE Activities**

[ Research Team]: *The last questions are about PSE activities that are priority areas for the state of Ohio. See handout 1. These have been identified by OSU/SNAP-Ed as key activities to target for preventing and reducing obesity among children in low-income populations.*

Healthy Eating PSEs in Food Pantries

1. Can you share more about your plans for conducting Healthy Eating PSEs in Food Pantries over the next 12 months? In your survey you mentioned [insert from survey].
2. Can you share more about how particular features of your county/community make healthy eating PSEs in food pantries easier or harder to implement?
3. Can you share more about how your work in food pantries is changing due to COVID-19?
4. **PSE READI**

*Our last questions are about the PSE READI website:* [*www.psereadi.org*](http://www.psereadi.org)*. This site has our current decision-making tools focused on farmers markets, farm to school, healthy eating policies in childcare, and healthy food retail.*

1. Do you have any feedback for us on the psereadi.org website?
2. Do you have recommendations for how we can enhance this site as we plan to introduce two new decision-making tools focused on healthy eating PSEs in healthy eating PSEs in food pantries?
3. What type of training and technical support can we offer to help you and other SNAP-Ed practitioners make use of the tools and resources on psereadi.org?

*That is all of the questions we have for you today. Before we wrap up, is there anything else you want to share with us about your interests and needs related to healthy eating activities in your community?*
